# Complete genome sequence of *Clostridium botulinum* type A strain (X58540) isolated from cattle feces

**DOI:** 10.1128/mra.00988-24

**Published:** 2025-02-24

**Authors:** Taeho Kim, Jin-Young Lee, Sukju Gil, Frank Gessler, Chang Hun Shin

**Affiliations:** 1 Research Institute, Chong Kun Dang Bio Co Ltd, Cheongju-si, South Korea; 2 miprolab GmbH, Göttingen, Germany; Portland State University, Portland, Oregon, USA

**Keywords:** *Clostridium botulinum*, genomes

## Abstract

The full genome sequence of *Clostridium botulinum* type A strain X58540 found in fecal samples from cattle exhibiting clinical symptoms of chronic botulism is presented here. The genome of X58540 consists of 2,371,631 bp with a GC content of 28.17% and is predicted to include 2,157 coding sequences.

## ANNOUNCEMENT

*Clostridium botulinum* strains are found in soil and water (1). The *C. botulinum* strain used in this study was isolated from fecal samples submitted to miprolab (Göttingen, Germany) in 2005 from cattle with chronic botulism on a farm in the northwestern region of Mecklenburg-Vorpommern, Germany.

The fecal samples were homogenized and incubated anaerobically in liquid-reinforced Clostridium medium (Oxoid, Germany) at 37°C for 4 days. Botulinum toxin was confirmed by intraperitoneal injection of culture supernatants and botulinum antitoxin-treated culture supernatants into mice following DIN 10102 (2). Samples with confirmed presence of botulinum toxin were streaked on egg yolk agar (3) and incubated anaerobically at 37°C for 2 days to verify the colony characteristics of botulinum (white, round, lecithinase+, and lipase+). Colonies exhibiting *C. botulinum* characteristics were subjected to PCR for botulinum neurotoxin genes using appropriate primers (4–6). A strain exhibiting the characteristics of *C. botulinum* type A was isolated and repeatedly cultured on egg yolk agar to obtain a pure culture designated as X58540.

Whole-genome *de novo* sequencing was performed using PacBio and Illumina systems on genomic DNA (gDNA) extracted from X58540 cultured anaerobically for 17 hours at 37°C in TPGY broth (MBcell, Korea) using the Wizard Genomic DNA Purification Kit (Promega, USA).

For PacBio sequencing, gDNA was sheared with g-TUBE (Covaris, USA) and purified using AMPurePB beads (Beckman Coulter, USA) to obtain 20 kb fragments. The library prepared using the SMRTbell Template Prep Kit 1.0 (PacBio, USA) was annealed using the PacBio DNA/Polymerase Binding Kit P6 (PacBio, USA). PacBio DNA sequencing kit 4.0 and eight SMRT cells (PacBio, USA) were used for sequencing on a PacBio RSII (PacBio, USA) with 240 min movies. Adapters were removed during the creation of subreads on the RSII platform. Assembly was performed through the SMRT portal (v2.3.0.140936; PacBio) with a minimum subread length of 500 bp, minimum polymerase read quality of 0.80, and minimum polymerase read length of 100 bp options. During the assembly, reads with a polymerase read sequencing quality <0.8 were removed, and after assembly, contig-level error correction was performed using Pilot (v1.21) (7). In total 127,352 reads were obtained with 1,222,525 bases, an average length of 9,599 bp, an N50 value of 13,407 bp, and a genome coverage of 157×.

For Illumina sequencing, the library prepared using the TruSeq PCR-free DNA High Throughput Library Prep Kit (Illumina, USA) was sequenced on a HiSeq2500 (Illumina, USA) with 101 bp paired-end reads. Quality control was performed using reads with >90% Q30 bases using Macrogen’s in-house scripts (Seoul, Korea). The genome coverage was 218×, and the total number of raw reads was 9,211,112 bp. The Unicycler (v. 0.4. 6) (8) was used for *de novo* assembly to automatically rotate the dnaA or repA genes to the initial position of the circular contig, trim the overlapping region, and ascertain whether the contig was circular. Genome annotation was performed using the NCBI Prokaryotic Genome Annotation Pipeline (PGAP) v5.0 (9–11). Genomic features are listed in Table 1.

**TABLE 1:** Characteristics of *Clostridium botulinum* type A strain X58540

Parameter	X58540
Length (bp)	3,815,652
GC (%)	28.17
Circular	Yes
Coding sequence	3,461
tRNA	80
rRNA (5S, 16S, 23S)	21 (7, 7, 7)

## Data Availability

The whole genome sequence was deposited in GenBank under accession number CP068960. The BioProject number is PRJNA693403, and the BioSample number is SAMN17386352. Raw reads were deposited in the NCBI Sequence Read Archive (SRA) under accession numbers SRX25324391 and SRX26602659 for the Illumina HiSeq2500 data and PacBio RSII data, respectively.
