# Correction for Kim et al., “Complete genome sequence of *Clostridium botulinum* type A strain (X58540) isolated from cattle feces”

**DOI:** 10.1128/mra.01273-25

**Published:** 2026-02-10

**Authors:** Taeho Kim, Jin-Young Lee, Sukju Gil, Frank Gessler, Chang Hun Shin

## AUTHOR CORRECTION

Volume 14, no. 3, e00988-24, 2025, https://doi.org/10.1128/mra.00988-24. In the Abstract, in the second sentence, “The genome of X58540 consists of 2,371,631 bp with a GC content of 28.17% and is predicted to include 2,157 coding sequences.” should read “The genome of X58540 consists of 3,815,652 bp with a GC content of 28.17% and is predicted to include 3,461 coding sequences.”

